# Trends and causes of maternal mortality in Ethiopia during 1990–2013: findings from the Global Burden of Diseases study 2013

**DOI:** 10.1186/s12889-017-4071-8

**Published:** 2017-02-02

**Authors:** Gizachew Assefa Tessema, Caroline O. Laurence, Yohannes Adama Melaku, Awoke Misganaw, Sintayehu A. Woldie, Abiye Hiruye, Azmeraw T. Amare, Yihunie Lakew, Berihun M. Zeleke, Amare Deribew

**Affiliations:** 1 Institute of Public Health, University of Gondar, Gondar, Ethiopia; 2 School of Public Health, The University of Adelaide, Adelaide, Australia; 3 Population Research and Outcome Studies, School of Medicine, The University of Adelaide, Adelaide, Australia; 4 School of Public Health, Mekelle University, Mekelle, Ethiopia; 5 Institute of Health Metrics and Evaluation, University of Washington, Seattle, USA; 6 Maternal and Child Health Directorate, Federal Ministry of Health, Addis Ababa, Ethiopia; 7 School of Medicine, The University of Adelaide, Adelaide, Australia; 8 College of Medicine and Health Sciences, Bahir Dar University, Bahir Dar, Ethiopia; 9 Department of Epidemiology, University of Groningen, Groningen, The Netherlands; 10 Ethiopian Public Health Association, Addis Ababa, Ethiopia; 11 Population KEMRI-Wellcome Trust Research Programme, Kilifi, Kenya; 12 Nuffield Department of Clinical Medicine, University of Oxford, Oxford, UK; 13 St. Paul Millennium Medical College, Addis Ababa, Ethiopia

**Keywords:** Maternal mortality, Trends, Global Burden of Diseases, Ethiopia

## Abstract

**Background:**

Maternal mortality is noticeably high in sub-Saharan African countries including Ethiopia. Continuous nationwide systematic evaluation and assessment of the problem helps to design appropriate policy and strategy in Ethiopia. This study aimed to investigate the trends and causes of maternal mortality in Ethiopia between 1990 and 2013.

**Methods:**

We used the Global Burden of Diseases and Risk factors (GBD) Study 2013 data that was collected from multiple sources at national and subnational levels. Spatio-temporal Gaussian Process Regression (ST-GPR) was applied to generate best estimates of maternal mortality with 95% Uncertainty Intervals (UI). Causes of death were measured using Cause of Death Ensemble modelling (CODEm). The modified UNAIDS EPP/SPECTRUM suite model was used to estimate HIV related maternal deaths.

**Results:**

In Ethiopia, a total of 16,740 (95% UI: 14,197, 19,271) maternal deaths occurred in 1990 whereas there were 15,234 (95% UI: 11,378, 19,871) maternal deaths occurred in 2013. This finding shows that Maternal Mortality Ratio (MMR) in Ethiopia was still high in the study period. There was a minimal but insignificant change of MMR over the last 23 years. The results revealed Ethiopia is below the target of Millennium Development Goals (MGDs) related to MMR. The top five causes of maternal mortality in 2013 were other direct maternal causes such as complications of anaesthesia, embolism (air, amniotic fluid, and blood clot), and the condition of peripartum cardiomyopathy (25.7%), complications of abortions (19.6%), maternal haemorrhage (12.2%), hypertensive disorders (10.3%), and maternal sepsis and other maternal infections such as influenza, malaria, tuberculosis, and hepatitis (9.6%). Most of the maternal mortality happened during the postpartum period and majority of the deaths occurred at the age group of 20–29 years. Overall trend showed that there was a decline from 708 per 100,000 live births in 1990 to 497 per 100,000 in 2013. The annual rate of change over these years was -1.6 (95% UI: -2.8 to -0.3).

**Conclusion:**

The findings of the study highlight the need for comprehensive efforts using multisectoral collaborations from stakeholders for reducing maternal mortality in Ethiopia. It is worthwhile for policies to focus on postpartum period.

## Background

Maternal health has become one of the major public health concerns for developing countries following the first safe motherhood conference held in Kenya in 1987 [1]. Yet, maternal mortality remains the global challenge with 275,288 deaths occurring due to pregnancy and complications in 2015 [2]. The Millennium Development Goal (MDG) set the target in 2000 in reducing maternal mortality by 75% for World Health Organization (WHO) member countries [3]. While some progress has been made, according to WHO estimate in 2015, the Maternal Mortality Ratio (MMR) dropping by 44% worldwide between 1990 and 2015 [3], it remains unacceptably high in developing countries particularly in sub-Sahara African countries [4, 5]. In one of these countries, Ethiopia, the MMR remains high, ranging from 266–1667 per 100,000 Live Births (LB) [3, 5–10].

Maternal mortality is the most sensitive indicator of the health disparities between poorer and richer nations, and for overall development. The effects of maternal mortality also have impacts on children and remaining families [11–15]. For instance, the infant and under-five survival is highly correlated with child nutrition and other important child health care practices demanding maternal involvement [12].

The causes of maternal mortality are multifactorial. An in-depth analysis on the trends of maternal health in Ethiopia pointed to demographic, behavioural, nutritional, and health services related factors are associated with poor maternal health outcomes [16]. Yet, the key factors attributable for the death of mothers are related to low facility deliveries, poor competence of providers, lack of emergency obstetric services at facilities, and inefficient referral systems for obstetric emergencies [17–20]. In this regard, several studies reported limited utilization of key maternal health services in Ethiopia [21–24]. The reasons for low maternal health services utilization were related to range of factors such as women’s sociodemographic factors, cultural factors, communal factors, limited access to health facilities, and poor quality of care in health facilities [25–28]. These complex and interlinked factors can be characterised by the three delays model [29]. The model comprises delay in deciding to seek care (delay 1), delay in reaching the health facility (delay 2), and delay in receiving quality care once at the health facility (delay 3). In dealing with the first delay, the government established Health Development Army (HDA) in 2010 with the aim of expanding the achievements of the Health Extension Programme (HEP) deeper into communities, improving community ownership and scaling up best practices [30]. In response to delay two, along with other strategies, the Ethiopian government introduced an innovative free ambulance services in providing ambulances in every rural district that can serve the communities on 24–hours, 7–days basis to transfer any woman in labour or experiencing other obstetric difficulties to the appropriate health facility [31]. Moreover, Maternal Death Surveillance and Response (MDSR) and Respectful Maternity Care (RMC) was launched to mitigate the challenges owing from delay in receiving quality maternal health services [32, 33]. As part of the Health Sector Transformation Plan (HSTP), Ethiopia aspires to reduce MMR to 177 death per 100,000 LB in 2020 [32]. Moreover, in the post-MDG era, the Sustainable Development Goal (SDG) puts an ambitious target of achieving MMR of 70 per 100,000 live births (LB) in 2030 [34]. Hence, in order to track future targets and assess the impact of government initiatives, understanding the past and present trends and causes of maternal mortality in Ethiopia is vital.

Measuring maternal mortality is difficult in low-income countries because of limited registration of births and deaths [35]. It becomes difficult as maternal mortality is relatively a rare event besides the challenge in avoiding the technical problems related to bias and the high demand of cost to carrying out sufficiently large surveys to measure the rate per unit time or per birth with reasonable precision [36, 37]. Most of the previous studies in Ethiopia were based on a single data sources, or sub-national study, or without identifying the causes of maternal mortality [6–8, 10, 38]. Unlike these studies, the GBD study provides a unique opportunity for its use of standardised methodology using several sources of data. This study used GBD study 2013 to investigate the trends and causes of maternal mortality between the years 1990 and 2013 in Ethiopia.

## Methods

### Study setting

Ethiopia is the second most populous country in Africa next to Nigeria with a projected total population of 98 million in 2016 and a total fertility rate of 4.1 children per women [39, 40]. The Ethiopian Demographic and Health Survey (EDHS) 2016 reported that 62% of the pregnant women used antenatal care, 28% women delivered with skilled attendance at birth, 17% received postnatal care, and 35% women practiced contraception with variations across regions. Moreover, this survey reported a MMR of 412 per 100,000 LB in 2016 [10]. Maternal health services are provided at all three level of the Ethiopian health system [32].

### Data sources and modelling

This study uses secondary data from GBD 2013 study [41]. The detailed methodological approach for the GBD study is published elsewhere [5, 42]. In summary, Kassabaum and Colleagues [5] reported that various data sources were explored including sibling histories from Demographic and Health Surveys (DHS), censuses, maternal mortality surveillance, and verbal autopsy analyses covering women of reproductive age. The International Classification of Diseases (ICD)-10 definition was regarded to account maternal death. *Maternal death* was defined as “the death of a woman while pregnant or within 42 days of termination of pregnancy, irrespective of duration and site of pregnancy from any cause related to or aggravated by the pregnancy and its management, but not from incidental or accidental causes” [43]^p98^. Deaths occurred between 42 days and the first year were taken as *late maternal death*. In GBD 2013 study, the underlying aetiologies assumed for *other direct maternal causes* were complications of anaesthesia, embolism (air, amniotic fluid, and blood clot), and the condition of peripartum cardiomyopathy.

HIV related mortality during pregnancy involved estimation of the fraction of deaths during pregnancy or within six weeks of delivery that are related to HIV, and estimation of the fraction of these HIV-related deaths that are aggravated by pregnancy. DerSimonian-Laird meta-analysis of the relative risk (RR) of death was undertaken [5]. The modified UNAIDS EPP/SPECTRUM suite model was used to estimate population attributable fraction of HIV-related deaths during pregnancy using the RR and estimated HIV prevalence [44].

The causes of death by maternal age were measured using the Cause of Death Ensemble model (CODEm) model. In the GBD study, the CODEm model was employed to group the covariate and enable computation. In the study, nine covariates that included age-specific fertility rate, total fertility rate, age-standardised HIV death rate for female individuals aged 15–49 years, neonatal death rate, lag-distributed gross domestic product (GDP) per person, proportion of deliveries occurring in facilities, proportion of deliveries attended by skilled birth attendants, coverage of four antenatal care visits, and malnutrition in children younger than 5 years (proxy for adult nutritional status) were undertaken based on their potential associations with maternal mortality [5]. Detail description of CODEm is reported elsewhere [44, 45]. Mixed effects linear regression and Spatio-temporal Gaussian Process Regression (ST-GPR) were applied while predicting the mortality rate and mortality fractions [45].

## Results

### Trends of maternal mortality in Ethiopia

**Fig. 1:**
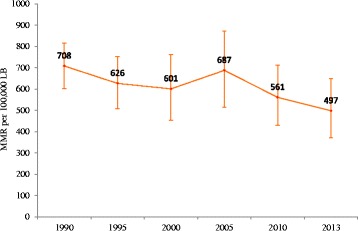
Trends of maternal mortality ratio in Ethiopia, 1990–2013

In Ethiopia, a total of 16,740 (95% UI: 14,197, 19,271) maternal deaths occurred in 1990 whereas there were 15,234 (95% UI: 11,378, 19,871) maternal deaths occurred in 2013. Overall trend showed that there was a decline from 708 per 100,000 LB in 1990 to 497 per 100,000 LB in 2013, with a small increase in 2005. The annual rate of change between 1990 and 2013 was -1.6 (95% UI -2.8 to -0.3) with no significant difference over the years (Table 1 and Fig. 1).

**Table 1:** Number of maternal mortality in Ethiopia 1990–2013

**Year**	**Number of deaths**	**Lower UI**	**Upper UI**
1990	16740	14197	19271
1991	16943	14349	19570
1992	17174	14369	19935
1993	17278	14159	20413
1994	17227	13952	20394
1995	17097	13877	20531
1996	16727	13345	20381
1997	16637	13067	20537
1998	16798	12988	20977
1999	16861	12783	21266
2000	17132	12934	21725
2001	17559	13045	22413
2002	18133	13504	23089
2003	18941	14001	24173
2004	19531	14534	24941
2005	19853	14838	25216
2006	19842	14880	25011
2007	19444	14776	24424
2008	18494	14230	23158
2009	17617	13475	22093
2010	16731	12835	21239
2011	15986	12183	20464
2012	15506	11778	20025
2013	15234	11378	19871

**Fig. 2:**
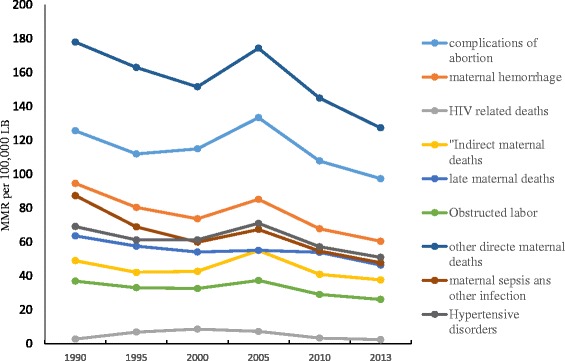
Trends of the causes of maternal mortality in Ethiopia, 1990–2013

The trends in maternal deaths due to other direct maternal causes such as complications of anaesthesia, embolism (air, amniotic fluid, and blood clot), and the condition of peripartum cardiomyopathy and complications of abortion seemed remarkable between 2005 and 2013 whereas deaths due to maternal haemorrhage, hypertensive disorder, and sepsis and other maternal infections showed smaller changes between 2005 and 2013. The trend in the last 23 years revealed that the contribution of obstructed labour followed similar pace. The death attributed to HIV infection was higher between 1995 and 2005 but later it significantly declined (Fig. 2).

### Causes of maternal mortality in Ethiopia

**Fig. 3:**
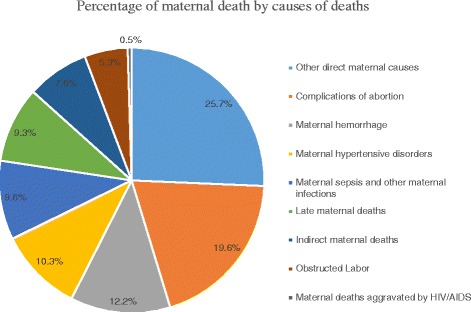
Percentage of maternal deaths by causes of maternal mortality in Ethiopia in 2013

The percentage of maternal deaths by causes of death in 2013 are shown in Fig. 3. The leading cause of maternal death that contributed more than three quarter of deaths in Ethiopia in 2013 was other direct maternal causes such as complications of anaesthesia, embolism (air, amniotic fluid, and blood clot), and the condition of peripartum cardiomyopathy (25.7%) followed by complications of abortions (19.6%), maternal haemorrhage (12.2%), hypertensive disorders (10.3%), and maternal sepsis and other maternal infections such as influenza, malaria, tuberculosis, and hepatitis (9.6%). However, maternal deaths related to HIV/AIDS attributed for 0.5% maternal deaths. This pattern was also seen in preceding years.

**Fig. 4:**
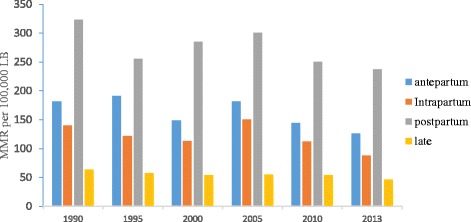
Trends of maternal mortality by timing of mortality, 1990–2013, Ethiopia

In terms of the timing of maternal death, the postpartum period accounted for the highest number of deaths over the last 23 years, whereas the late postpartum period (42 days to one year) accounted the least number of maternal deaths. In 2013, there were 237 maternal deaths per 100,000 LB during postpartum period while the MMR during antepartum, intrapartum and late periods were 126, 88, and 46 per 100,000LB respectively (Fig. 4).

**Fig. 5:**
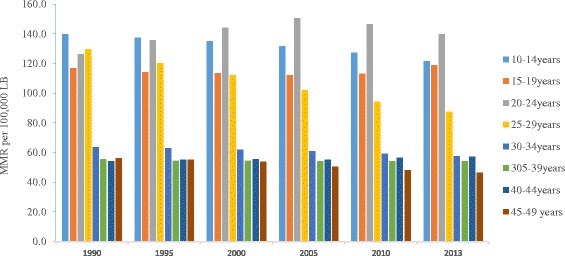
Trends of maternal mortality by maternal age, Ethiopia, 1990–2013

The ratios of maternal mortality also varied across maternal age groups. Over the different time periods since 1990, the highest rate of maternal mortality as measured through MMR occurred in the age group 20 and 39 years. In the year 2005, the MMR was highest in the age group 20–24 years whereas it became highest among the 25–29 age group from 2010 onwards. Unlike in 1990, the MMR was reduced among 30–35 years 35–39 age groups in 2013. Over the last 23 years, maternal mortality occurred among the young adolescent girls 10–14 age group (Fig. 5).

## Discussion

This study found that maternal mortality in Ethiopia over the period 1990–2013 was considerably high and remained above the Millennium Development target goal set in 2000. The 2013 MMR estimate for this study (497 per 100,000LB) showed that while there was a decrease in MMR in the previous two decades, the decline was not significant. The finding based on the GBD data was higher than that estimated by WHO [46] (353 per 100,000LB), and reports in Northern Ethiopia [7] (266 per 100 000 LB), Southwest Ethiopia (425 per 100,000LB) [6]; however, it was lower than that reported by the EDHS 2016 report (412 per 100,000) [10], and a study from Southern Ethiopia [8] (1667 per 100,000 LB). The differences with the present findings and studies in northern and southwest Ethiopia might be due to the differences in the sample size of study population and the sources of data, and maternal mortality estimation methodology. For instance, while the GBD 2013 estimate employed several sources of data, the studies in the northern and southwest Ethiopia were solely based on verbal autopsy. The difference between the GBD 2013 estimate and the WHO estimate was due to several reasons [47]. Firstly, GBD 2013 involved many site-years of data while the WHO estimate included subnational data from urban and rural areas. Secondly, there was a difference in assumptions while addressing stochastic fluctuation and small numbers. Thirdly, it could be attributed from the time period differences between the two estimates. The differences between the present findings and the EDHS 2016 report could be likely due to the time differences although the later report relied on small number of events (maternal deaths) occurred in 7- years preceding the survey [10].

The annual rate of decline by -1.6% (95% UI: -2.8 to -0.3) was far lower than the targeted annual decline (5.5%) to achieve the MDG five goal. This minimal change in maternal mortality is likely due to the high proportion of deliveries occurred at home. In this regard, the EDHS report between 2000 and 2016, showed that only 5–28% of deliveries occurred in health facilities [10, 38, 40, 48, 49]. This is suggestive of the need for rigorous efforts in terms of improving facility delivery and women’s access to emergency obstetric care services.

This study found that the major causes of maternal mortality were other direct maternal causes such as anaesthesia, embolism (air, amniotic fluid, and blood clot), and the condition of peripartum cardiomyopathy followed by complications of abortion. This is inconsistent with two reviews which reported that abortion related complications and obstructed labor/uterine rupture accounted for the top leading causes of maternal mortality in Ethiopia [19, 50]. The differences in the present finding and the previous reviews is likely to be due to the inclusion of hospital-based studies. As a result, this may have led to an overestimation in some of the causes of deaths, with obstructed deaths and complications of abortions were more likely to be reported for health facilities.

This study found that mortality due to HIV related causes was relatively stable with a small decline, over time. This reduction may have resulted from the introduction of the Prevention of Mother-To-Child Transmission (PMTCT) of HIV/AIDS services since 2001 [51]. A study conducted in Ethiopia also suggested that there was a remarkable improvement in terms of potential coverage of PMTCT services between 2006 and 2010 [52]. Furthermore, the national HIV prevalence has shown a decline in the general population including pregnant women [53].

As reported by other previous studies [5, 54], a higher number of deaths occurred during postpartum period over the study period. The occurrence of a higher number of deaths in this period could be due to two main reasons. Firstly, it could be due to the unpredictability of complications and the necessity to advanced lifesaving services to lessen these complications during delivery and the immediate postpartum period. The second reason could be due to lack of quality obstetric services at health facilities for women attending facility delivery. For instance, research suggests that health providers in Ethiopia may have limited competency in terms of managing postpartum haemorrhage and utilization of partograph for monitoring labour progress [55, 56]. Another study from Northwest Ethiopia also reported the lack of basic signal functions necessary for routine and emergency maternity care services [57]. This suggests the need to enhance accessibility of emergency obstetric care to women in Ethiopia. A recent study conducted in Wolisso district of Ethiopia reported that no deaths occurred amongst mothers who used the ambulance services to reach facilities providing emergency obstetric services [58]. In this respect, the roles of Health Extension Workers (HEW) and Women’s Developmental Army (WDA) were vital in terms of organising women’s use of skilled birth attendance by arranging ambulance services and timely referral of labouring mothers to health facilities [59].

The present study also showed that although the decline in MMR varied across age groups, with a higher number of maternal deaths among the age groups of 20–24 and 25–29 years old in 2013. This finding likely reflects the high fertility rate in these age groups of women and thus the potential of increased maternal mortality [40]. In this regard, the EDHS 2016 report pointed to high rates of maternal mortality in these specific age categories [10]. The higher rate of young adolescents of 10–14 years are dying over the study period could be associated with high prevalence of early marriage and unmet need for family planning services. This suggests that programs that aim to reduce pregnancy amongst young adolescents could reduce the risk of maternal mortality arising from childbirth complications due to gynaecological immaturity and incomplete pelvic growth.

The present study has strengths due to use of robust methodology and multiple sources of data that enabled us to see the overtime trends of maternal mortality in Ethiopia. However, the study had some limitations. Firstly, as there was no vital registration system in Ethiopia, the modelling was based on limited sources of data, which may influence the results. Secondly, as indicated in the large gaps in the 95% uncertainty intervals, the estimate was made on relatively smaller sample size. Thirdly, while it was possible to see the mortality differences across different age groups, the present estimate could not show the MMR in terms of other women’s sociodemographic status such as urban-rural residence, wealth and educational status.

## Conclusion

While there has been a reduction in the MMR in Ethiopia over the last 23 years, the annual rate of reduction is far lower than targets set. The MMR trends observed in 2013 showed that Ethiopia did not achieve the MDG target of reducing MMR to 267 per 100,000LB. In 2013, the top five causes of maternal mortality included other direct maternal causes such as anaesthesia, embolism (air, amniotic fluid, and blood clot), and the condition of peripartum cardiomyopathy, complications of abortions, maternal haemorrhage, hypertensive disorders, and maternal sepsis and other maternal infections such as Influenza, malaria, tuberculosis and hepatitis. Most of the maternal mortality occurred during the postpartum period and majority of the deaths occurred at the age groups of 20–25 and 25–29 years in 2013. The findings of the study highlights the need for comprehensive efforts using multisectoral collaborations from stakeholders in reducing maternal mortality in Ethiopia.
